# Factors influencing early and long-term survival following hip fracture among nonagenarians

**DOI:** 10.1186/s13018-021-02807-6

**Published:** 2021-10-30

**Authors:** Laurence Weinberg, Bobby Ou Yang, Luka Cosic, Sarah Klink, Peter Le, Jasun Kai Li, Anoop Ninan Koshy, Daryl Jones, Rinaldo Bellomo, Chong Oon Tan, Dong-Kyu Lee

**Affiliations:** 1 Department of Anaesthesia, Austin Health, 145 Studley Rd, Melbourne, VIC 3084 Australia; 2 Department of Surgery, The University of Melbourne, Austin Health, Melbourne, VIC 3084 Australia; 3 Department of Cardiology, Austin Health, Melbourne, VIC 3084 Australia; 4 Department of Intensive Care, Austin Health, Melbourne, VIC 3084 Australia; 5 Department of Critical Care, The University of Melbourne, Melbourne, VIC 3084 Australia; 6 Department of Anesthesiology and Pain Medicine, Dongguk University Ilsan Hospital, Ilsandong-gu, Goyang, 10326 Republic of Korea

**Keywords:** Anaesthesia, Complication, Nonagenarian, Fracture, Surgery

## Abstract

**Background:**

The outcomes of nonagenarian patients undergoing orthopaedic surgery are not well understood. We investigated the 30-day mortality after surgical treatment of unilateral hip fracture. The relationship between postoperative complications and mortality was evaluated.

**Methods:**

We performed a single-centre retrospective cohort study of nonagenarian patients undergoing hip fracture surgery over a 6-year period. Postoperative complications were graded according to the Clavien–Dindo classification. Correlation analyses were performed to evaluate the relationship between mortality and pre-specified mortality risk predictors. Survival analyses were assessed using Cox proportional hazards regression modelling.

**Results:**

The study included 537 patients. The 30-day mortality rate was 7.4%. The mortality rate over a median follow-up period of 30 months was 18.2%. Postoperative complications were observed in 459 (85.5%) patients. Both the number and severity of complications were related to mortality (*p* < 0.001). Compared to patients who survived, deceased patients were more frail (*p* = 0.034), were at higher ASA risk (*p* = 0.010) and were more likely to have preoperative congestive heart failure (*p* < 0.001). The adjusted hazard ratio for mortality according to the number of complications was 1.3 (95% CI 1.1, 1.5; *p* = 0.003). Up to 21 days from admission, any increase in complication severity was associated significantly greater mortality [adjusted hazard ratio: 3.0 (95% CI 2.4, 3.6; *p* < 0.001)].

**Conclusion:**

In a nonagenarian cohort of patients undergoing hip fracture surgery, 30-day mortality was 7.4%, but 30-month mortality rates approached one in five patients. Postoperative complications were independently associated with a higher mortality, particularly when occurring early.

**Supplementary Information:**

The online version contains supplementary material available at 10.1186/s13018-021-02807-6.

## Introduction

In both absolute numbers, and as a proportion of many Western populations, the number of people in their eighth, ninth and tenth decades continues to increase [1, 2]. Hip fractures are similarly becoming more common and carry an associated increased burden of morbidity and mortality [1–3]. In Australia, hip fracture-related hospital admissions account for approximately 0.5% of all hospitalisations and result in more than 579,000 bed days or 1.9% of annual hospitalisations [4]. Despite reductions in hospitalisation rates among ageing and at-risk populations with hip fractures, these patients still present a significant clinical burden [4]. Hip fractures are over-represented in the elderly, with a median age of hospitalisation in Australia being 84 years [4]. When compared to younger populations, nonagenarians (patients aged 90–99 years) are highly vulnerable to hip fractures and experience increased rates of postoperative morbidity, mortality and adverse functional outcomes [2, 5, 6].

Surgical osteosynthesis is considered the gold standard of care for hip fracture, with nearly all patients receiving operative management in Australia [4]. While some international literature has reported on the preoperative, surgical and anaesthetic factors affecting postoperative outcomes in nonagenarian patients [2, 7, 8], no comparative data exist in the Australian context. Moreover, the majority of existing data focus on preoperative factors affecting post-surgical outcomes, including time to surgery, patient comorbidity and advanced age [2, 7, 8]. Additionally, there remains a dearth of international data assessing the impact of complications and perioperative factors on short- and long-term mortality among nonagenarians.

Given the strong association between increasing age and mortality secondary to hip fractures [5, 9, 10], and the significant burden that these injuries place on healthcare systems, it is valuable to identify perioperative factors that may present opportunities to reduce morbidity and mortality among nonagenarians suffering from them. Therefore, we investigated the overall mortality in patients who, in their tenth decade, underwent surgery for hip fracture. The primary outcome was 30-day mortality. Secondary outcomes included the number and severity of postoperative complications and their association with short- and long-term mortality.

## Methods

Following Human Research Ethics Committee approval (No: HREC/21/Austin/30), we performed a single-centre retrospective cohort study of nonagenarian patients who underwent post-fracture restorative surgery over a six-year period between 1 September 2014 and 31 August 2020. Perioperative outcomes for the period ending 28 February 2021 were collected, providing a minimum of 6-month postoperative data following the final surgical episode. The study was performed at the Austin Hospital, a tertiary university teaching hospital in Melbourne, Australia. Inclusion criteria were patients aged > 90 years and < 100 years presenting with a unilateral femoral neck, intertrochanteric or subtrochanteric femoral fracture (collectively termed ‘hip fracture’), who required operative management. All operative techniques were considered. Patients were excluded if they were managed conservatively or died prior to operative management. Four independent investigators extracted data from the electronic medical records. The above dates were chosen a priori because Austin Health Cerner^®^ electronic medical records allowed comprehensive electronic data capture and retrieval of patient health information in the perioperative setting from September 2014 onwards.

### Objectives

The primary endpoint of this study was to quantify the rate of perioperative mortality at 30 postoperative days. Secondary endpoints were the number and severity of postoperative complications and their association with short- and long-term mortality. We also evaluated the perioperative risk factors associated with long-term survival. Mortality was assessed using Austin Health’s electronic medical record system or by contacting patients’ general practitioners to obtain out-of-hospital mortality status.

### Definitions

Medical comorbidities were collected from electronic medical records, including anaesthetic records. Charlson comorbidity index (CCI) scores were calculated for each patient [11]. Frailty was defined as a score of 5 or more on the Canadian Study of Health and Ageing Clinical Frailty Scale [12]. Time to surgery was defined as the length of time from hospital presentation until the commencement of anaesthesia. Surgery performed out-of-hours was defined as surgery performed between 6 p.m. and 8 a.m. on Monday to Friday, and on Saturdays, Sundays and public holidays. Return to theatre was defined as any second or subsequent surgery performed as a consequence of the original surgical management of hip fracture during the index admission. Complications were defined as any deviation from the normal postoperative course, guided by the European Perioperative Clinical Outcome definitions [13]. Complications were recorded by two independent clinicians and graded according to the Clavien–Dindo (CVD) classification [14]. The CVD classification is a validated approach to surgical outcome assessment that assigns severity grades to surgical complications. In case of disagreement on grading by two assessors, the case was decided with reference to the classification guide by a third assessor. Instances of review by the hospital Medical Emergency Team (MET) were obtained from the dedicated electronic database, completed by the intensive care registrar at the conclusion of the MET call. Length of stay was determined by the period between presentation and discharge. Readmission was defined as an unplanned readmission to the hospital within a 30-day follow-up period.

### Statistical analysis

Statistical analysis was performed using R software 4.0.2 (R Development Core Team, Vienna, Austria, 2020). Data were deidentified, the variable names were encrypted, and data were coded with numerical values to blind the collected variables’ characteristics to the statistician. Data are presented as mean ± standard deviation, median (1st–3rd quartiles) [Max/Min], or the number of cases (percentile) for descriptive statistics. Estimated values are described with 95% confidence intervals (CI). Statistical results are presented with *p* values. Any two-sided *p* value below 0.05 or Bonferroni’s corrected significance levels was considered statistically significant.

Before statistical analysis, normality was assessed for continuous variables using the quantile–quantile plot. If normality criteria were violated, nonparametric statistical methods were applied for that variable. The homogeneity of variance assumption was applied where appropriate. Standard statistical methods identified extreme values, which were then reconciled by interrogating the clinical notes and the context of the value.

Variables with a missing rate of more than 5% were identified, and missing value patterns were analysed to determine missingness mechanisms. Data that were missing completely at random were excluded from the statistical analysis. Student’s *t* test, Mann–Whitney rank-sum test, Chi-squared test and Fisher’s exact test were applied to determine the differences between survived and deceased cases. Correlation analysis was performed to evaluate the relationship between mortality and other variables. According to these results and knowledge based on their clinical impacts, several factors were identified as independent parameters for the following survival analysis. Considering the longitudinal characteristics, Cox proportional hazard regression modelling was used for survival analysis. The goodness-of-fit test for constant proportional hazard assumption was assessed using Schoenfeld residuals. In case of violated constant proportional hazard assumption, time-dependent Cox regression was applied according to the split observation duration using a step function. The splitting point was determined by the interpretation of Schoenfeld residuals.

## Results

### Details of cohort

The final analysis included data from 537 patients. There were two variables with a missing value rate of more than 5%: height (51.2%) and preoperative albumin concentration (35.2%). The pattern of omission for both was random, but because neither variable carried a statistically significant relationship to mortality, these omissions were tolerated. Over the study follow-up of 30 (12–53) [1:77] months, 98 deaths were recorded (18.2%, 95% CI 15.0, 21.5%). Of the cases of mortality, 40 patients (7.4%, 95% CI 5.2, 9.7%) died within 30 days from admission.

### Baseline associations with 30-day mortality

Baseline patient characteristics are presented in Table 1 and in Additional file 1. Several associations with mortality were identified. Males made up 25% of all patients and 36.7% of all deceased patients (*p* = 0.019); however, the effect of sex differences was small (Cramér’s *V* = 0.10). The age difference between surviving and deceased patients was not statistically significant. Frailty was associated with a higher unadjusted mortality rate, although the estimated effect size was small. Finally, deceased patients had higher ASA classification scores (*p* = 0.010) and a higher incidence of congestive heart failure on admission (*p* < 0.001).

**Table 1:** Demographic data for nonagenarian patients undergoing surgery for hip fracture

Category and variables	Survived (*n* = 439)	Deceased (*n* = 98)	*p* value	Effect size	Correlation coefficient (*p* value)
Demographics					
Sex (Male)^+^	110 (25.1)	36 (36.7)	0.019*	0.10	0.101 (0.019)*
Age (years)^‡^	92.97 ± 2.70	93.26 ± 2.61	0.339	0.11	0.056 (0.197)
Frailty^+^	327 (74.7)	83 (84.7)	0.034*	0.09	0.091 (0.034)*
Weight (kg)^‡^	59.23 ± 11.93	61.68 ± 12.39	0.068	0.20	0.071 (0.101)
ASA classification^§^					
II	40 (9.1)	5 (5.1)	0.010*	–	0.135 (0.002)*
III	248 (56.5)	43 (43.9)			
IV	150 (34.2)	49 (50.0)			
V	1 (0.2)	1 (1.0)			
Diabetes mellitus^+^	73 (16.6)	19 (19.4)	0.512	0.03	0.028 (0.513)
Chronic kidney disease^+^	111 (25.3)	30 (30.6)	0.279	0.05	0.047 (0.279)
Congestive heart failure^+^	97 (22.1)	40 (40.8)	< 0.001*	0.17	0.166 (< 0.001)*
Chronic obstructive airways disease^+^	44 (10)	15 (15.3)	0.130	0.07	0.065 (0.131)
Cerebrovascular accident/transient ischaemic attack^+^	84 (19.1)	13 (13.3)	0.172	0.06	–0.059 (0.173)
Dementia^+^	166 (37.8)	39 (39.8)	0.715	0.02	0.016 (0.716)
Charlson comorbidity index	6 (5–7) [4:14]	6 (5–8) [4:15]	0.297	–0.05	0.045 (0.297)

Values are expressed as mean ± SD, median (IQR) [Max/Min], or number (%)

Effect size: Cohen’s *d* for *t* test, common effect size *r* for Mann–Whitney test, Cramér’s *V* for the Chi-squared test. Correlation coefficient: Spearman’s rho and corresponding *p* value. Time to surgery: time from admission to surgery start. Opioid doses are presented as a total amount of all kinds of opioids used as a morphine-equipotent dose

*ASA classification* American Society of Anesthesiologist physical status classification, *CCI* Charlson’s comorbidity index

*Two-sided *p* value < 0.050

^+^Chi-squared test

^‡^T test

^§^Fisher’s exact test

^¶^Mann–Whitney test

### Perioperative associations with 30-day mortality

As shown in Table 2, preoperative and intraoperative differences between survivors and deceased patients were small and did not reveal any clear risk factor. The postoperative course, however, was characterised by a higher frequency of medical emergency team (MET) calls in deceased patients (*p* = 0.019, common effect size *r* = –0.10).

**Table 2:** Surgical management, perioperative and anaesthetic clinical information amongst nonagenarian patients undergoing surgery for hip fracture

Category and variables	Survived (*n* = 439)		Deceased (*n* = 98)	*p* value		Effect size		Correlation coefficient (*p* value)	
Surgical factors									
Time to surgery from admission to hospital (hours)^‡^	34.25 ± 37.49		38.28 ± 45.92	0.358		0.10		0.042 (0.328)	
Operation time (min)^‡^	131.92 ± 137.71		138.77 ± 145.15	0.660		0.05		− 0.004 (0.921)	
Surgery performed out-of-hours^+^	237 (54)		61 (62.2)	0.137		0.06		0.064 (0.137)	
Emergency^§^	432 (98.4)		95 (96.9)	0.400		–		− 0.042 (0.332)	
Femur neck fracture^+^	367 (83.6)		78 (79.6)	0.341		0.04		− 0.041 (0.342)	
Preoperative block^+^	235 (53.5)		59 (60.2)	0.230		0.05		0.052 (0.231)	
Combined other surgery^+^	25 (5.7)		8 (8.2)	0.358		0.04		0.040 (0.358)	
Preoperative conditions									
Preoperative transfusion^+^	23 (5.2)		5 (5.1)	0.956		0.001		− 0.002 (0.956)	
Anaesthesia factors									
Regional anaesthesia combined^+^	200 (45.6)	45 (45.9)			0.948		0.003		0.003 (0.948)
Invasive monitoring^+^	311 (71.2)	73 (74.5)			0.509		0.03		0.029 (0.510)
Intraoperative vasopressors used^+^	371 (84.5)	87 (88.8)			0.281		0.05		0.047 (0.282)
Intraoperative fluid management									
Total fluid amount (ml)^¶^	1000 (1000–1000) [0:3000]	1000 (1000–1000) [0:2250]			0.729		− 0.01		0.015 (0.729)
No. of patients who received an intraoperative transfusion^+^	23 (5.2)	9 (9.2)			0.136		0.06		0.064 (0.136)
Intraoperatively transfused red blood cell units	1 (1‒1) [1:2], N = 23	1 (1–1.5) [1:3], N = 9			0.273		− 0.194		–
No. of events of Intraoperative hypotension^¶^	0 (0–2) [0:21]	0 (0–1) [0:13]			0.038*		− 0.09		− 0.090 (0.038)*
No. of events of Intraoperative hypotension, severe	0 (0–0) [0:28]	0 (0–0) [0:16]			0.246		− 0.05		− 0.050 (0.246)
Opioid									
Preoperative opioid used^+^	323 (73.6)	79 (80.6)			0.147		0.06		0.063 (0.147)
Intraoperative opioid use^+^	352 (80.2)	85 (86.7)			0.132		0.07		0.065 (0.132)
Intraoperative opioid dose (mg)^‡^	10.1 ± 12.57	12.88 ± 21.19			0.087		0.19		0.039 (0.365)
Postoperative opioid used^+^	172 (39.2)	43 (44.8)			0.310		0.04		0.044 (0.311)
Patient controlled analgesia^+^	131 (29.8)	24 (25)			0.344		0.04		− 0.041 (0.345)
Postoperative opioid dose (mg)^‡^	183.59 ± 448.78		323.52 ± 788.17	0.096		0.27		0.052 (0.228)	
Postoperative management									
Postoperative hypotension episodes	0 (0–1) [0:26]		0 (0–0) [0:15]	0.362		− 0.04		− 0.039 (0.362)	
Postoperative vasopressor use^+^	31 (7.1)		10 (10.3)	0.276		0.05		0.047 (0.277)	
ICU care^§^	12 (2.7)		4 (4.1)	0.509		–		0.031 (0.479)	
Return to theatre^+^	20 (4.6)		8 (8.2)	0.146		0.06		0.063 (0.147)	
Readmission^+^	11 (2.5)		5 (5.1)	0.187		0.06		0.059 (0.172)	
No. of medical emergency team activations^¶^	0 (0–0) [0:7]		0 (0–1) [0:5]	0.019*		− 0.10		0.101 (0.019)*	

Values are expressed as mean ± SD, median (IQR) [Max/Min], or number (%)

Effect size: Cohen’s *d* for *t* test, common effect size *r* for Mann–Whitney test, Cramér’s *V* for the Chi-squared test. Correlation coefficient: Spearman’s rho and corresponding *p* value. Time to surgery: time from admission to surgery start. Opioid doses are presented as a total amount of all kinds of opioids used as a morphine-equipotent dose

*Two-sided *p* value < 0.050

^+^Chi-squared test

^‡^*T* test

^§^Fisher’s exact test

^¶^Mann–Whitney test

### Association between postoperative complications and mortality

As shown in Table 3, both the number and severity of postoperative complications were associated with overall mortality (*p* < 0.001). Complications were observed in 459 patients (85.5%, 95% CI 82.5, 88.0). The observed complication rate was 84.5% (95% CI 81.1, 87.9) in surviving patients and 89.8% (95% CI 83.8, 95.8) in deceased patients. The number of postoperative complications was higher in the deceased compared to surviving patients (*p* < 0.001). Similarly, the severity of complications was higher in deceased patients (*p* < 0.001, Table 3). Gender, frailty, ASA classification, congestive heart failure, pre-anaesthesia hypertension, the number of intraoperative hypotensive episodes, the number of MET calls and the number or severity of complications were selected covariates for the Cox regression. In addition to these, weight and intra- and postoperative opioid dose were included in the analysis as covariates in view of their traditional statistical threshold as potential predictors [15]. Given their clinical importance, CCI [11], time to surgery from hospital admission [16], surgery performed out-of-hours [17, 18] and combined regional anaesthesia [19] were also considered covariates for the subsequent survival analysis.

**Table 3:** Postoperative complications in surviving and deceased patients

Complications	Survived (*n* = 439)	Deceased (*n* = 98)	*p* value	Common effect size *r*	Correlation coefficient (*p* value)
Number of complications					
No complication	68 (15.5)	10 (10.2)	< 0.001*	0.19	0.16 (< 0.001)*
1 complication	85 (19.4)	15 (15.3)			
2 complications	98 (22.3)	10 (10.2)			
3 complications	66 (15.0)	15 (15.3)			
4 or more complications	122 (27.8)	48 (49.0)			
Clavien Dindo grade					
No complication	68 (15.5)	10 (10.2)	< 0.001*	0.49	0.22 (< 0.001)*
I	92 (21.0)	17 (17.3)			
II	248 (56.5)	35 (35.7)			
IIIa	6 (1.4)	2 (2.0)			
IIIb	15 (3.4)	3 (3.1)			
IVa	9 (2.1)	4 (4.1)			
IVb	1 (0.2)	0 (0.0)			
V	0 (0.0)	27 (27.6)			

Cochran–Armitage test for trend and Spearman correlation analysis

*Two-sided *p* value < 0.025

Cox regression was performed separately for the number or severity of complications. The proportional hazard assumption was violated in both cases of the number and severity of complications. According to the Schoenfeld residuals, the observed period was split at 12 days after admission for the number of complications and at 21 days and 17 months after admission for the severity of complications. A time-dependent coefficient Cox regression was then used to analyse overall mortality with respect to the remaining covariates.

**Fig. 1:**
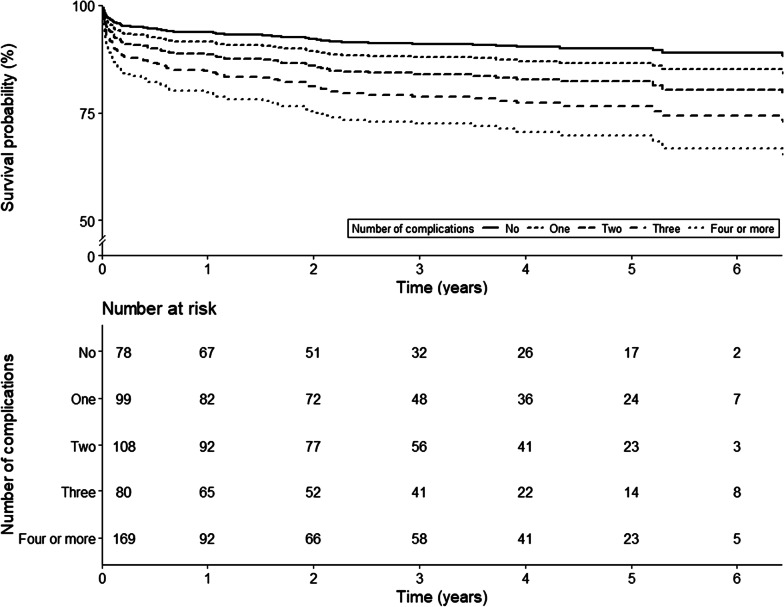
Survival curves according to the number of complications in nonagenarian hip surgery patients using time-dependent coefficient Cox regression

**Fig. 2:**
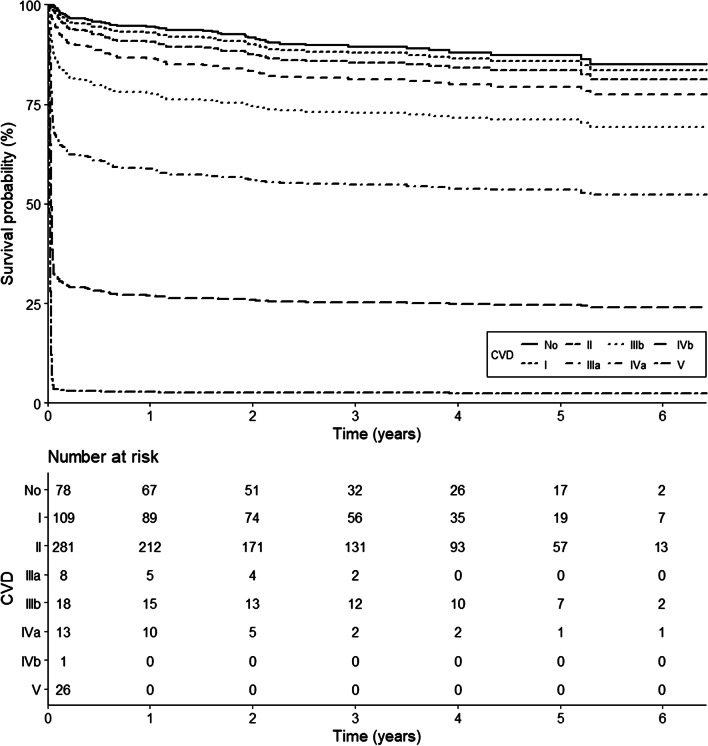
Survival curves according to the Clavien–Dindo grade of complications in nonagenarian hip surgery patients using time-dependent coefficient Cox regression

As shown in Table 4 and Figs. 1 and 2, the adjusted hazard ratio for the number of complications was 1.3 (95% CI 1.1, 1.5; *p* = 0.003) during the observed period for these patients (Fig. 1). Increasing Clavien–Dindo grade of complication was associated with increased adjusted risk of mortality (HR 3.0 (95% CI 2.4, 3.6; *p* < 0.001 per 1 unit rise in CVD) until 21 days from admission (Fig. 2). After 21 days, the effects of postoperative complications on mortality were not statistically significant. A summary of the postoperative complications is presented in Additional file 2.

**Table 4:** Estimated hazard ratios of selected covariates for overall mortality in nonagenarian hip surgery patients

	No. of complications		Severity of complications	
	Hazard ratio	*p* value	Hazard ratio	*p* value
Sex	1.4 (0.9–2.2)	0.197	1.5 (0.9–2.4)	0.095
Frailty	1.5 (0.8–2.6)	0.174	1.4 (0.8–2.5)	0.241
Weight	1.0 (1.0–1.0)	0.791	1.0 (1.0–1.0)	0.490
ASA classification	1.3 (0.5–3.4)	0.534	1.2 (0.5–3.0)	0.731
Congestive heart failure	1.8 (1.2–2.8)	0.008*	1.7 (1.1–2.7)	0.027
Charlson comorbidity index	1.0 (0.9–1.2)	0.640	1.0 (0.9–1.2)	0.524
Time to surgery	1.0 (1.0–1.0)	0.391	1.0 (1.0–1.0)	0.154
Surgery performed out-of-hours	1.5 (1.0–2.2)	0.084	1.7 (1.1–2.6)	0.017*
Hypertensive response immediately before anaesthesia induction	1.7 (0.9–3.3)	0.093	1.1 (0.5–2.3)	0.773
Combined regional anaesthesia	1.1 (0.7–1.7)	0.557	1.2 (0.8–1.9)	0.378
Number of intraoperative hypotensive episodes	0.9 (0.8–1.0)	0.039	0.9 (0.8–1.0)	0.116
Intraoperative opioid dose	1.0 (1.0–1.0)	0.119	1.0 (1.0–1.0)	0.206
Postoperative opioid dose				
Day of admission to 12 days	1.0 (1.0–1.0)	0.265	1.0 (1.0–1.0)	0.006*
After 12 days	1.0 (1.0–1.0)	0.005*		
No. of medical emergency team activations	1.0 (0.8–1.3)	0.802	0.9 (0.7–1.2)	0.609
Number of complications	1.3 (1.1–1.5)	0.003*	–	–
Clavien Dindo severity				
Day of admission to 12 days	–	–	3.0 (2.4–3.6)	< 0.001*
12 days to 7 months	–	–	1.2 (0.9–1.6)	0.145
After 7 months	–	–	1.0 (0.6–1.6)	0.982

Hazard ratios are estimated using constant proportional hazard ratio assumption validated Cox regression for number of complications and time-dependent coefficient Cox regression for severity of complications. Estimated hazard ratios are presented with 95% CI

*Two-sided *p* value below 0.025, a Bonferroni’s corrected significance level

## Discussion

### Key findings

We investigated the short-term and long-term outcome of nonagenarians undergoing hip fracture surgery and assessed the relationship of complications with mortality in this cohort [1, 3, 20]. We found that short-term mortality affected one in fifteen patients and that long-term mortality affected one in five patients. Moreover, we also found that preoperative and intraoperative characteristics and events did not show a clear association with outcome. However, we also found that postoperative complications affected almost 90% of patients and that their number and severity were associated with increased risk of death.

### Relationship to literature

Preoperative optimisation remains a challenge in this cohort. We did not identify time to surgery as an independent risk factor for increased postoperative mortality. The average time to surgery in our cohort was 34 h in patients who survived and 38 h in deceased patients. These times are aligned with international guidelines [21], systematic reviews, meta-analysis, meta-regression [16, 22–24] and large database registries [25, 26], which demonstrate reduced mortality and intraoperative complications if hospitals operate on patients within 48 h after fracture [27].

Some studies have also reported that surgery performed after hours significantly increases general complication rates [27]. Potential contributing factor for these increased risks include surgeon fatigue, surgeon experience, and the potential for more severe or emergent injuries occurring after-hours. Other large-scale studies have refuted such findings [28]. In a systematic review and meta-analysis of 13 studies with 177,090 patients, Kim et al. reported that performing hip fracture surgery after hours or on the weekend did not increase the risk of 30-day or inpatient mortality or postoperative complications, and that consideration should be given to performing hip fracture surgery out-of-hours to meet national guidelines (< 48 h) [28]. In the present study, we did not observe any significant differences in mortality when surgery was performed out-of-hours. Possible reasons for this finding are that our institution has a dedicated ‘out-of-hours’ consultant-led emergency orthopaedic service that facilitates operating room availability and allows early and timely access to surgery. The overall mortality rate for nonagenarians having hip fracture surgery at our institution was comparable to that reported in the previous literature [2, 3, 29].

There is disagreement within the literature regarding the impact of comorbidity among nonagenarians on mortality. Some studies [3, 29] corroborate our findings, while others [2, 3, 5, 20, 30, 31] have reported increasing comorbidity to be associated with increased mortality. Our findings indicate postoperative complications have the greatest significance regarding mortality following hip fracture surgery.

### Study implications

Our findings imply that short-term mortality after hip surgery in nonagenarians is relatively low. However, it also implies that, as expected, once follow-up is extended to 30 months, one in five such patients have died. It also implies that preoperative characteristics and intraoperative events are not major risk factor for such postoperative mortality. However, it implies that postoperative complications are extremely common and are a major risk factor for mortality, especially when occurring in the early postoperative period.

### Strengths and limitations

Our study presents new data regarding the impact of complications on mortality among nonagenarians having hip fracture surgery. The Clavien–Dindo classification allowed us to demonstrate a close correlation between complication severity and increasing mortality for the study cohort. The extended follow-up time frames also allowed us to quantify the period for which surgical complications increased the risk of post-surgical mortality. We acknowledge several limitations of this study. The retrospective design inherently limits the quality of its findings. Despite this, the missing data points were small in number and did not statistically affect our findings. Given that data were collected retrospectively, the follow-up period varied for patients depending on the year of surgery. This may have affected the long-term mortality data because, at the time of data collection, patients with more recent surgeries had shorter postoperative timeframes. This may have led to an underestimation of mortality; however, the large sample size mitigates this effect. Data on the causes of delays in receiving surgery were not collected. A better understanding of these details may provide further insights into why this variable had no impact on mortality despite previous studies being strongly suggestive of such an effect.

The CCI has been shown to effectively predict mortality following hip fracture; however, the index only collects data on selected comorbidities [31]. This specificity of the CCI may explain why our findings did not identify comorbidity as a predictor of mortality. We did not assess functional outcomes. Functional outcomes in the nonagenarian cohort are arguably more important than objective measures of mortality, and future studies may be enhanced by assessing functional outcome measures such as the World Health Organisation Disability Assessment Schedule (WHODAS).

## Conclusion

Patients aged between 90 and 99 years undergoing elective or emergent hip fracture surgery had a 30-day mortality rate of 7.4% and mortality within 30 months after surgery approached 20%. Almost nine out of ten patients developed at least one postoperative complication. The development of any postoperative complication was independently associated with a higher mortality throughout the entire observation period, especially when they occurred in the earlier postoperative period. Our findings suggest that strategies to minimise postoperative complications may improve postoperative survival in this patient age group. Further study and efforts to reduce complications among nonagenarians having hip fracture surgery are warranted.

## Supplementary Information

**Additional file 1.** Detailed demographic data, laboratory findings and clinical information for nonagenarian patients with a hip fracture undergoing surgical treatment.

**Additional file 2.** The number of postoperative complications after hip fracture surgery among nonagenarians. Most patients had more than one complication.

## Data Availability

The complete deidentified dataset analysed during the current study is available from the corresponding author on reasonable request.

## Abbreviations

ASA: American Society of Anesthesiologists
CI: Confidence interval
CVD: Clavien–Dindo
HR: Hazard ratio
MET: Medical Emergency Team
WHODAS: World Health Organisation Disability Assessment Schedule
