# Supplementary material for: Factors influencing early and long-term survival following hip fracture among nonagenarians
Source: J Orthop Surg Res. 2021 Oct 30;16:653. doi: 10.1186/s13018-021-02807-6 (PMC8557574; doi:10.1186/s13018-021-02807-6)
Supplement: Supplementary file 1 — Additional file 1. Detailed demographic data, laboratory findings and clinical information for nonagenarian patients with a hip fracture undergoing surgical treatment. [file 13018_2021_2807_MOESM1_ESM.docx]

## Supplementary Table 1. Detailed demographic data, laboratory findings and clinical information for nonagenarian patients with a hip fracture undergoing surgical treatment

| **Category and variables** | | | **Survived (N = 439)** | **Deceased (N = 98)** | **P value** | **Effect size** | **Correlation coefficient (P value)** |
| --- | --- | --- | --- | --- | --- | --- | --- |
| **Demographics** | | | | | | | |
| Preoperative albumin (g/L)‡ | | | 32.33 ± 5.60 | 31.13 ± 5.13 | 0.119 | 0.217 | –0.069 (0.196) |
| Preoperative haemoglobin (g/dL)‡ | | | 117.54 ± 18.16 | 115.72 ± 19.03 | 0.377 | 0.099 | –0.033 (0.441) |
| Preoperative creatinine (*u*mol/L)‡ | | | 92.69 ± 35.34 | 114.12 ± 55.54 | <0.001* | 0.539 | 0.175 (<0.001*) |
| Diabetes mellitus+ | | Patients with no diabetic related complications | 46 (10.5) | 10 (10.2) | 0.936 | 0.003 | –0.003 (0.936) |
|  |  | Patients with diabetic related complications + | 27 (6.2) | 9 (9.2) | 0.278 | 0.047 | 0.047 (0.278) |
| Myocardial ischaemia+ | | | 100 (22.8) | 28 (28.6) | 0.224 | 0.053 | 0.053 (0.224) |
| Malignancy without metastasis+ | | | 64 (14.6) | 10 (10.2) | 0.256 | 0.049 | –0.049 (0.257) |
| Malignancy with metastasis^§^ | | | 8 (1.8) | 4 (4.1) | 0.245 | - | 0.059 (0.172) |
| Peripheral vascular disease+ | | | 26 (5.9) | 4 (4.1) | 0.473 | 0.031 | –0.031 (0.474) |
| Hemiplegia^§^ | | | 4 (0.9) | 2 (2.0) | 0.301 | - | 0.042 (0.337) |
| Connective tissue disease^§^ | | | 10 (2.3) | 2 (2.0) | >0.999 | - | –0.006 (0.884) |
| Peptic Ulcer disease^§^ | | | 13 (3) | 3 (3.1) | >0.999 | - | 0.002 (0.958) |
| Total Hip Replacement^§^ | | | 14 (3.2) | 2 (2.0) | 0.748 | - | –0.026 (0.546) |
| Partial Hip Replacement+ | | | 127 (28.9) | 27 (27.6) | 0.785 | 0.012 | –0.012 (0.785) |
| Gamma Nailing+ | | | 51 (11.6) | 11 (10.2) | 0.912 | 0.005 | –0.005 (0.913) |
| Medullary Rod+ | | | 60 (13.7) | 15 (15.3) | 0.672 | 0.018 | 0.018 (0.673) |
| Dynamic Hip Screw+ | | | 103 (23.5) | 18 (18.4) | 0.275 | 0.047 | –0.047 (0.276) |
| Other types of hip surgery+ | | | 19 (4.3) | 9 (9.2) | 0.051 | 0.084 | 0.084 (0.051) |
| **Preoperative haemodynamic variables** | | | | | | | |
| Preoperative systolic blood pressure‡ | | | 141.79 ± 21.87 | 134.16 ± 20.54 | 0.002* | 0.353 | –0.13 (0.002)* |
| Preoperative diastolic blood pressure‡ | | | 71.96 ± 10.82 | 71.25 ± 11.58 | 0.559 | 0.065 | –0.044 (0.311) |
| Preoperative mean blood pressure‡ | | | 95.27 ± 12.47 | 91.98 ± 12.45 | 0.018* | 0.264 | –0.103 (0.017)* |
| Preoperative systolic hypertension+ | | | 189 (43.1) | 24 (24.5) | 0.001* | 0.147 | –0.147 (0.001)* |
| **Anaesthetic variables** | | | | | | | |
| General anaesthesia, intravenous+ | | | 45 (10.3) | 11 (11.2) | 0.775 | 0.012 | 0.012 (0.776) |
| General anaesthesia, volatile+ | | | 287 (65.4) | 67 (68.4) | 0.572 | 0.024 | 0.024 (0.573) |
| Regional anaesthesia combined+ | | Spinal anaesthesia combined+ | 126 (28.7) | 20 (20.4) | 0.095 | 0.072 | –0.072 (0.096) |
|  |  | Epidural anaesthesia combined^§^ | 1 (0.2) | 2 (2.0) | 0.087 | - | 0.094 (0.029)* |
|  |  | Regional block combined+ | 109 (24.8) | 34 (34.7) | 0.046 | 0.086 | 0.086 (0.046)* |
| Airway management+ | | No tracheal intubation | 140 (32.0) | 23 (23.5) | 0.087 | 0.095 | 0.039 (0.362) |
|  |  | Tracheal intubation performed | 251 (57.3) | 68 (69.4) |  |  |  |
|  |  | Supraglottic airway device | 47 (10.7) | 7 (7.1) |  |  |  |
| **Intraoperative management** | | | | | | | |
| No. of intraoperative systolic hypotension | | | 0 (0 ‒ 2) [0:29] | 0 (0 ‒ 3) [0:22] | 0.314 | –0.043 | –0.044 (0.314) |
| No. of intraoperative diastolic hypotension | | | 0 (0 ‒ 3) [0:34] | 0 (0 ‒ 3) [0:26] | 0.464 | –0.032 | –0.032 (0.465) |
| **Postoperative management** | | | | | | | |
| Postoperative diastolic hypotension | | | 1 (0 ‒ 3) [0:16] | 0 (0 ‒ 3) [0:20] | 0.290 | –0.046 | –0.046 (0.290) |
| Postoperative systolic hypotension | | | 0 (0 ‒ 0) [0:8] | 0 (0 ‒ 0) [0:7] | 0.915 | –0.005 | 0.005 (0.915) |
| Transfer after surgery^§^ | | General ward | 418 (95.2) | 89 (91.8) | 0.072 | - | 0.061 (0.158) |
|  |  | High dependency unit | 17 (3.9) | 4 (4.1) |  |  |  |
|  |  | Intensive care unit | 4 (0.9) | 4 (4.1) |  |  |  |
| **Medical Emergency Team data** | | | | | | | |
| Code blue^§^ (cardiac or respiratory arrest) | | | 7 (1.6) | 5 (5.1) | 0.081 | 0.075 | 0.091 (0.034)* |
| **Medical Emergency Team data** | | | | | | | |
| Number of MET calls¶ | | | 0 (0 ‒ 0) [0:7] | 0 (0 ‒ 1) [0:5] | 0.020 | –0.101 | 0.101 (0.019)* |
|  | Tachycardia | | 0 (0 ‒ 0) [0:3] | 0 (0 ‒ 0) [0:3] | 0.519 | –0.028 | –0.028 (0.520) |
|  | Bradycardia | | 0 (0 ‒ 0) [0:0] | 0 (0 ‒ 0) [0:0] | >0.999 | 0.000 | - |
|  | Hypertension | | 0 (0 ‒ 0) [0:2] | 0 (0 ‒ 0) [0:0] | 0.412 | –0.035 | –0.035 (0.413) |
|  | Hypotension | | 0 (0 ‒ 0) [0:3] | 0 (0 ‒ 0) [0:3] | 0.049* | –0.085 | 0.085 (0.049)* |
|  | High respiration rate | | 0 (0 ‒ 0) [0:3] | 0 (0 ‒ 0) [0:3] | 0.043* | –0.087 | 0.088 (0.043)* |
|  | Low respiration rate | | 0 (0 ‒ 0) [0:1] | 0 (0 ‒ 0) [0:0] | 0.412 | –0.035 | –0.035 (0.413) |
|  | Low oxygen saturation | | 0 (0 ‒ 0) [0:4] | 0 (0 ‒ 0) [0:2] | 0.001* | –0.143 | 0.143 (0.001)* |
|  | Low urine output | | 0 (0 ‒ 0) [0:1] | 0 (0 ‒ 0) [0:1] | 0.015* | –0.105 | 0.105 (0.015)* |
|  | Staff worried about patient | | 0 (0 ‒ 0) [0:1] | 0 (0 ‒ 0) [0:1] | 0.920 | –0.004 | –0.004 (0.920) |
|  | Low Glasgow Coma score | | 0 (0 ‒ 0) [0:3] | 0 (0 ‒ 0) [0:2] | 0.203 | –0.055 | 0.055 (0.204) |
|  | Severe uncontrolled pain | | 0 (0 ‒ 0) [0:1] | 0 (0 ‒ 0) [0:0] | 0.637 | –0.02 | –0.020 (0.637) |
|  | Breathing difficulty | | 0 (0 ‒ 0) [0:1] | 0 (0 ‒ 0) [0:1] | 0.030* | –0.094 | 0.094 (0.029)* |
|  | Other | | 0 (0 ‒ 0) [0:1] | 0 (0 ‒ 0) [0:1] | 0.156 | –0.061 | 0.061 (0.156) |

Patients are grouped by the overall mortality.

Values are expressed as mean ± SD, median (IQR) [Max:Min], or number (%).

*: 2-sided P value < 0.050 or P value < 0.0038 (Bonferroni’s correction for multiple comparison)

+: Chi-squared test

‡: t-test

§: Fisher’s exact test

¶: Mann-Whitney test

ASA classification: American Society of Anesthesiologist physical status classification, CCI: Charlson’s comorbidity index, GCS: Glasgow coma scale. Effect size: Cohen’s d for t-test, common effect size r for Mann-Whitney test, Cramér’s V for the chi-squared test. Correlation coefficient: Spearman’s rho and corresponding P value. Time to surgery: time from admission to surgery start.
