# Supplementary material for: Factors influencing early and long-term survival following hip fracture among nonagenarians
Source: J Orthop Surg Res. 2021 Oct 30;16:653. doi: 10.1186/s13018-021-02807-6 (PMC8557574; doi:10.1186/s13018-021-02807-6)
Supplement: Supplementary file 2 — Additional file 2. The number of postoperative complications after hip fracture surgery among nonagenarians. Most patients had more than one complication. [file 13018_2021_2807_MOESM2_ESM.docx]

## Supplementary Table 2. The number of postoperative complications after hip fracture surgery among nonagenarians. Most patients had more than 1 complication.

|  | **Total patients**  **N=537** | **Patients who died within 30 days of index surgery**  **N=40** | **Patients who died over the median study follow-up period of 30 months**  **N=98** |
| --- | --- | --- | --- |
| **Cardiac** | | | |
| Myocardial infarction or myocardial injury after non cardiac surgery | 19 (3.5%) | 5 (12.5%) | 6 (6.1%) |
| Congestive cardiac failure including pulmonary oedema | 80 (14.9%) | 15 (37.5%) | 29 (29.6%) |
| Hypotension (volume depletion or vasoplegia requiring treatment) | 89 (16.6%) | 10 (25.0%) | 22 (22.4%) |
| Arrhythmia | 60 (11.2%) | 4 (10.0%) | 12 (12.2%) |
| Cardiopulmonary arrest | 2 (0.4%) | 1 (2.5%) | 0 (0.0%) |
| Other (e.g., bradycardia requiring treatment, syncope, pericarditis) | 39 (7.3%) | 0 (0.0%) | 5 (5.1%) |
| **Total** | **289** | **35** | **74** |
| **Haematological** | | | |
| Anaemia | 172 (32.0%) | 10 (25%) | 25 (25.5%) |
| Thrombosis/embolism (excluding pulmonary) or coagulopathy | 6 (1.1%) | 1 (2.5%) | 2 (2.0%) |
| Other (e.g., febrile neutropaenia) | 4 (0.7%) | 0 (0.0%) | 0 (0.0%) |
| **Total** | **182** | **11** | **27** |
| **Pulmonary** | | | |
| Respiratory failure or respiratory distress syndrome | 11 (2.0%) | 2 (5.0%) | 40 (4.1%) |
| Pneumonia including aspiration | 82 (15.3%) | 10 (25.0%) | 24 (24.5%) |
| Pulmonary embolism | 9 (1.7%) | 0 (0.0%) | 2 (2.0%) |
| Other (e.g., pleurisy, cough, pneumothorax) | 15 (2.8%) | 2 (5.0) | 6 (6.1%) |
| **Total** | **117** | **14** | **72** |
| **Gastrointestinal** | | | |
| Haemorrhage | 9 (1.7%) | 2 (5.0%) | 5 (5.1%) |
| Diarrhoea | 10 (1.9%) | 0 (0.0%) | 2 (2.0%) |
| Constipation requiring intervention | 108 (20.1%) | 5 (12.5%) | 19 (19.4%) |
| Other (e.g., high stoma output, pancreatitis, bowel obstruction) | 36 (6.7%) | 4 (10.0%) | 9 (9.2%) |
| **Total** | **163** | **11** | **35** |
| **Infective** | | | |
| Systemic inflammatory response syndrome or sepsis | 21 (3.9%) | 7 (17.5%) | 10 (10.2%) |
| Line or cannula infection | 14 (2.6%) | 0 (0.0%) | 0 (0.0%) |
| Wound infection | 7 (1.3%) | 0 (0.0%) | 4 (4.1%) |
| **Total** | **42** | **7** | **14** |
| **Metabolic and endocrine** | | | |
| Electrolyte disturbance requiring intervention | 69 (12.8%) | 9 (22.5%) | 21 (21.4%) |
| Acid base derangements requiring intervention | 5 (0.9%) | 1 (2.5%) | 1 (1.0%) |
| Blood sugar complication (e.g., hypoglycaemia, diabetic ketoacidosis | 15 (2.8%) | 2 (5.0%) | 2 (2.0%) |
| Other (e.g. nutritional deficiency) | 7 (1.3%) | 0 (0.0%) | 3 (3.1%) |
| **Total** | **96** | **12** | **27** |
| **Neurological** | | | |
| Stroke or transient ischemia attack | 8 (18.6%) | 1 (2.5%) | 4 (4.1%) |
| Delirium including confusion and hallucinations | 175 (32.6%) | 16 (40%) | 37 (37.8%) |
| Other (e.g., headache, encephalopathy, psychiatric illness) | 21 (3.95) | 2 (5.0%) | 6 (6.1%) |
| **Total** | **204** | **19** | **47** |
| **Renal** | | | |
| Acute kidney injury | 79 (14.7%) | 10 (25.0%) | 20 (20.4%) |
| Urinary tract infection | 56 (10.4%) | 1 (2.5%) | 5 (5.1%) |
| Urinary retention | 45 (8.4%) | 4 (10.0%) | 9 (9.2%) |
| Other (e.g., polyuria, orchitis, scrotal oedema, vaginal bleeding) | 31 (5.8%) | 2 (5.0%) | 7 (7.1%) |
| **Total** | **211** | **17** | **41** |
| **Dermatological** | | | |
| Pressure sore | 23 (4.3%) | 1 (2.5%) | 9 (9.2%) |
| Other (e.g., urticaria, rash) | 11 (2.0%) | 0 (0.0%) | 3 (3.1%) |
| **Total** | **34** | **1** | **12** |
| **Other** | | | |
| Haemorrhage or haematoma | 11 (2.0%) | 0 (0.0%) | 2 (2.0%) |
| Implant dislocation or failure | 7 (1.4%) | 1 (2.5%) | 3 (3.1%) |
| Other surgical complication | 23 (4.3%) | 0 (0.0%) | 8 (8.2%) |
| Uncontrolled pain | 27 (5.0%) | 2 (5.0%) | 5 (5.1%) |
| Mechanical fall | 30 (5.6%) | 2 (5.0%) | 11 (11.2%) |
| Other non-surgical cause not listed (e.g., medication error, alcohol withdrawal, drug reaction, musculoskeletal) | 52 (9.7%) | 17 (42.5%) | 16 (16.3%) |
| **Total** | **150** | **22** | **45** |
| **Grand total** | **1488** | **149** | **394** |

Complications were defined by as any deviation from the normal postoperative course or guided by the European Perioperative Clinical Outcome definitions.^13^

*Cardiac*

**Arrhythmia:** Arrhythmia is defined as electrocardiograph (ECG) evidence of cardiac rhythm disturbance.

**Cardiogenic pulmonary oedema:** evidence of fluid accumulation in the alveoli due to poor cardiac function.

**Myocardial infarction:** Increase in serum cardiac biomarker values with at least one value above the 99th percentile upper reference limit and at least one of the following criteria: symptoms of ischaemia; new or presumed new significant ST segment or T wave ECG changes or new left bundle branch block.

**Myocardial injury after non-cardiac surgery:** Peak troponin T (TnT) 0.03 ng ml/l judged due to myocardial ischaemia (i.e., no evidence of a nonischaemic aetiology causing the TnT elevation).

*Respiratory*

**Respiratory failure:** Postoperative PaO_2_ <8 kPa (60 mmHg) on room air, a PaO_2_:FI0_2_ ratio <90% and requiring oxygen therapy.

**Pleural effusion:** Chest radiograph demonstrating blunting of the costophrenic angle, loss of sharp silhouette of the ipsilateral hemidiaphragm in upright position, evidence of displacement of adjacent anatomical structures or (in supine position) a hazy opacity in one hemithorax with preserved vascular shadows.

**Atelectasis:** Lung opacification with a shift of the mediastinum, hilum or hemidiaphragm toward the affected area, and compensatory over-inflation in the adjacent non-atelectatic lung.

**Pneumothorax:** Air in the pleural space with no vascular bed surrounding the visceral pleura Bronchospasm Newly detected expiratory wheezing treated with bronchodilators.

**Aspiration pneumonitis:** Acute lung injury after the inhalation of regurgitated gastric contents.

**Respiratory Distress Syndrome:** The Berlin definition of respiratory distress syndrome.

**Pulmonary embolism:** A new blood clot or thrombus within the pulmonary arterial system.

*Renal*

**Acute Kidney Injury:** Kidney Disease Improving Global Outcomes (KDIGO) guidelines.

**Urinary tract infection:** a positive urine culture of 105 colony forming units/ml with no more than two species of micro-organisms, and with at least one of the following symptoms or signs: fever (>38.8^o^C), urgency, frequency, dysuria, suprapubic tenderness, costovertebral angle pain or tenderness with no other recognised cause.

*Infection*

**Infection:** meeting two or more of the following criteria: core temperature < 36^o^C or >38^o^C; white cell count >12x10^9^/l or < 4 x10^9^/l, respiratory rate >20 breaths per minute or PaCO_2_ < 4.7 kPa (35 mmHg); pulse rate >90 beats per minute.

**Surgical site infection (superficial)** (1) Infection occurs within 30 days after surgery and (2) Involves only skin and subcutaneous tissue of the incision and (3) The patient has at least one of the following: (a) purulent drainage from the superficial incision (b) organisms isolated from an aseptically obtained culture of fluid or tissue from the superficial incision (c) at least one of the following symptoms or signs of infection: pain or tenderness, localised swelling, redness or heat, and superficial incision is deliberately opened by surgeon and is culture positive or not cultured.

**Surgical site infection (deep):** Infection occurs within 30 days after surgery if no implant is left in place or 1 year if implant is in place. (2) Involves deep soft tissues (e.g., fascial and muscle layers) of the incision. (3) The patient has at least one of the following: (a) purulent drainage from the deep incision but not from the organ/space component of the surgical site (b) a deep incision spontaneously dehisces or is deliberately opened by a surgeon and is culture-positive or not cultured when the patient has at least one of the following symptoms or signs: fever (>38.8^o^C), or localised pain or tenderness.

*Other*

**Anaemia:** World Health Organisation criteria - haemoglobin <130 g/l in males and <120 g/l in females.

**Deep vein thrombosis:** A new blood clot or thrombus within the venous system.

**Delirium:** identified using the intensive care delirium screening checklist.

**Gastrointestinal bleed:** clinical or endoscopic evidence of blood in the gastrointestinal tract.

**Paralytic ileus:** Failure to tolerate solid food or defecate for three or more days after surgery.
